# More than the sum of its parts—A constructivist grounded-theory study on specialist palliative care during crises like the COVID pandemic

**DOI:** 10.1177/02692163231222771

**Published:** 2024-01-16

**Authors:** Julia Wikert, Claudia Bausewein, Farina Hodiamont

**Affiliations:** Department of Palliative Medicine, LMU University Hospital, Munich, Germany

**Keywords:** Palliative care, specialist palliative care, crisis, COVID-19, pandemic, complexity, qualitative research, grounded theory

## Abstract

**Background:**

The COVID pandemic is an example of a crisis challenging healthcare systems worldwide. The impact of the pandemic on providing high-quality palliative care calls for a deeper understanding of specialist services during crises. This is essential in preparation for further crises.

**Aim:**

To develop a conceptual understanding of the impact of the pandemic on specialist palliative care as an example for arising future crises.

**Design:**

Qualitative interview study across Germany, following a constructivist grounded theory methodology.

**Setting/participants:**

Eleven semi-structured interviews with experts with overarching knowledge of structures and processes in specialist palliative care between 05–07/2020 and between 02–06/2021, 23 semi-structured interviews with healthcare professionals working in a specialist palliative care setting.

**Results:**

The complex system of palliative care provision during crises has properties that cannot be understood as separated parts of the care process. The pandemic led to unique structural and processual challenges characterized by interconnectedness, uncertainty, dynamic, underlying dilemmas, and unclear long-term goal. In response to the pandemic, teams experienced different phases, which enhanced adaption, innovation, and progress within complex care situations. Creative strategy approaches and dynamic responsiveness facilitated innovative development and could lead to long-lasting improvement within services. Availability of information, transparent communication, comprehensible instructions, participation in decision-making, and search for solutions contributed to teams’ proactive development throughout the pandemic.

**Conclusion:**

Addressing the complex problems in specialist palliative care caused by crises requires system thinking and a learning mindset. This can facilitate teams to overcome the crisis and move forward rather than bounce back to normal.

**What is already known about the topic?**

- Specialist palliative care services are severely affected by crises like the COVID-19 pandemic.
- The multilayered impact of crises on specialist palliative care requires a conceptualization and cannot be understood by descriptive studies alone.
- The COVID pandemic will likely only be an example of several expected future crises affecting healthcare worldwide and calls for preparedness.

**What this paper adds?**

- Interconnectedness, uncertainty, dynamics, underlying dilemmas, and unclear long-term goals characterize the impact of crises and increase the complexity of specialist palliative care.
- Despite resulting challenges, crises can facilitate the development and improvement of services in flexible, adaptive teams.
- System thinking and collaborative decision-making contribute to the successful design and organization of services in crisis times.

**Implications for practice, theory, or policy**

- Specialist palliative care structures should be open to flexible and dynamic reactions under changing circumstances during crises and allow for a trial-and-error mentality.
- Preparing for disruptions and collaborative decision-making within the multidisciplinary team should be ongoing.
- Changes triggered by the pandemic should be reflected to sustain and further develop improvements for services to move forward.

## Background

Specialist palliative care is a fundamentally important part of healthcare and gains even more relevance in crises to ensure appropriate care and support.^1^ Healthcare professionals face unique challenges during crises, such as limited resources and high patient volumes, demonstrated impressively by the COVID-19 pandemic.^2^

Beyond that, it is well-known that care situations in palliative care need to be considered as complex problems shaped by reciprocal, nonlinear relations.^3^ The rapid spread of the coronavirus has further increased the challenges of specialist palliative care and led to persistently extensive countermeasures.^4^ Decision-making in specialist palliative care requires a systemic view to understand and address the complexity of the care situation^3^ and to continue providing the best possible care. Crises increase this premise.

While the principles of palliative care do not change during a crisis, specific adaptations seem unavoidable as an additional layer of complexity is added to the complex system of specialist palliative care.^5^ Staff shortages, impeded communication, visiting and contact restrictions^6^ up to the complete closure of palliative care units^7^ since the early course of the COVID pandemic are examples of substantial problems in providing adequate palliative care in crisis times. Furthermore, previous studies identified organizational responses by specialist palliative care teams, for example, increasing outreach or initiating telehealth approaches where possible.^5^ Specialist palliative care can be regarded as a key player in managing the impact of healthcare crises.^1^ Therefore, it remains highly relevant to not only identify and describe single effects but also understand how the system of specialist palliative care as a whole manages crises like a pandemic and overcomes related challenges. Furthermore, palliative care interventions are neglected in critical situations, mainly explained by the limited understanding and appreciation of palliative care in diverse critical situations.^8^ Previous research on the impact of crises on specialist services has descriptively addressed what was going on but often lacked the underlying how and why. A comprehensive, conceptual, in-depth analysis of specialist palliative care during crises like the pandemic is missing. Exemplified with experiences in Germany, we therefore sought to develop an integrated understanding of specialist palliative care during the COVID-19 pandemic, which is transferable to other crises.

## Methods

### Design

We conducted a qualitative interview study based on a constructivist grounded theory methodology^9,10^ as the research question required a flexible, qualitative approach to develop a theoretical understanding of patterns and effects related to the topic, especially possible changes evoked by severe crises. We triangulated two qualitative data sources (a, b) to include the meso-level and micro-level points of view.

Dataset (a) originated from expert interviews nested in the national research project COMPANION.^11,12^ These interviews primarily focused on structure and process characteristics for a typology of specialist palliative care, which has been analyzed and reported elsewhere.^12^ Due to the outbreak of COVID-19 shortly before data collection with a non-negligible impact on health care, we complemented the interview guide. We included further questions on the current pandemic, which interviewees discussed on a meso-level of care. All data material related to this additional focus has not been used elsewhere and is subject to primary analysis in this study.

Dataset (b) complemented the required information for a comprehensive understanding of the practical effects on specialist palliative care as perceived by individuals who provided direct patient care during the pandemic.

To ensure a high scientific standard the Consolidated criteria for reporting qualitative studies (COREQ) guideline was followed for reporting.^13^

### Setting

This was a national study conducted in specialist palliative care. In Germany, specialist palliative care is provided by multiprofessional teams on palliative care units, palliative care advisory teams in hospitals and specialist palliative home care teams in the community.

### Population

The study population included experts in specialist palliative care and healthcare professionals of various disciplines (see Table 1 for inclusion and exclusion criteria). Participants were (a) experts with overarching knowledge of structures and processes in specialist palliative care and (b) healthcare professionals working in a German specialist palliative care setting (palliative care unit, palliative care advisory team, specialist palliative home care).

**Table 1.** Participant inclusion and exclusion criteria.

	Experts	Healthcare professionals
Inclusion criteria	● Engagement in professional associations and/or other political commitments related to palliative care ● At least 4 years of respective work experience ● Overarching expertise regarding structures and processes in at least one adult specialist palliative care setting	● Currently working in an adult specialist palliative care setting ● At least 3 years of palliative care experience
Exclusion criteria	● Currently not being actively involved in specialist palliative care development	● Not working in specialist palliative care before the onset of the pandemic ● Currently working in other types of specialist palliative care services (e.g., children)

### Sampling

Consistent with constructivist grounded theory, purposive sampling was used initially and further developed throughout data collection and analysis as an iterative, ongoing process. This included consideration of multidisciplinary perspectives and variation regarding age, gender, and profession. Concerning the COVID situation and participants’ exposure, we included participants with relevant experience from various settings as well as different geographical and demographical areas, including those with higher and lower COVID incidences. Theoretical sampling continued until saturation was reached.^9^

### Recruitment

Participants were approached via e-mail by a member of the research team. The research team and the German Association for Palliative Medicine suggested experts. Healthcare professionals were identified through professional contacts of the research team and team leads of various specialist palliative care teams across Germany. Participants were known professionally to the authors in a few cases before the study.

### Data collection

The study took place during two significant phases of the COVID-19 pandemic. The expert interviews for dataset (a) were conducted during the summer plateau of 2020, and the interviews with healthcare professionals (b) during the third wave of infections in 2021. These pandemic phases were defined and published retrospectively by the Robert Koch Institute (RKI), Germany’s central biomedical institution.^14^

a) Semi-structured expert interviews were conducted face-to-face or via videoconferencing between May and July 2020 using a topic guide (see Appendix 1). In the interviews, participants were asked to discuss what has changed in the structures and processes of specialist palliative care since the outbreak of COVID-19.
b) Semi-structured episodic interviews with healthcare professionals were conducted via videoconferencing following a topic guide between February and June 2021 (see Appendix 2) The rationale for the methodological choice of episodic interviews was the distinction between episodic and semantic knowledge.^15^ While the latter comprises more generalized, decontextualized knowledge and perspectives without specific links to events or situations, episodic knowledge refers to situational knowledge linked to specific circumstances.^15^ The focus on lived experience within concrete circumstances linked to specific situations made episodic interviews most suitable to combine the widely-used semi-structured interview with narrative parts. We followed the phases suggested for episodic interviews by Flick.^16^ After introducing the participants to the features and course of episodic interviewing, interviewees were invited to elaborate openly on their associations with the COVID-19 pandemic. Subsequently, the participants were asked to talk about their experiences with the outbreak and the impact of the pandemic on their work routines in specialist palliative care by recalling situations and stories (episodic knowledge). Eventually, to gain insight into the semantic knowledge, we asked about fundamental values and meaningful aspects of participants’ professional roles and explored links to the pandemic impact.

All interviews were audio recorded and transcribed verbatim without any identifying data.

### Analysis

#### Expert interviews

Interview transcripts and field notes were analyzed by qualitative content analysis.^17,18^ Codes were developed discursively and included inductive and deductive coding. Transcripts were reviewed and discussed continually by a multi-professional research team to enhance the confirmability and dependability of the results.^19^ MAXQDA 2022 facilitated data management.^20^

#### Episodic interviews

In line with constructivist grounded theory, the analysis of the episodic interviews followed a gradual approach.^10^ Initially, focused and axial coding were included in the analysis as suggested by Charmaz^9^: After openly exploring and labeling all verbatim transcripts either line-by-line, incident by incident, and/or situation by situation (initial coding), initial codes were revised and conceptually refined with a focus on linkage, context, and category development (focused coding) (see Appendix 3). The axial coding phase focused on the broader picture by developing highly refined themes and theoretical links and specifying the dimensions of a category to attain the emerging theory.

#### Triangulation

During the analysis of the different perspectives, specific connections between identified categories and patterns became evident. To systematically triangulate the findings, we combined and merged the results from both data sets (expert and episodic interviews) on an increasingly abstract and contrasting level. Therefore, we compared patterns within the data sets for similarities and differences, as suggested by Flick.^21^

Analytical memos were used as an additional tool to enhance reflection.^22^ These notes became additional data to capture comparisons and explore ideas as a pivotal element of the analytic process.^9,22^ To increase the trustworthiness of the results, member checking^23^ was applied by confirming emergent findings with participants for accuracy.

### Ethical considerations

Ethical approval was received from the Local Research Ethics Committee of Ludwig-Maximilians-University Munich [ID 19-864 for dataset (a), and 20-422 for dataset (b)]. All participants provided written informed consent to the study and were advised about their right to refuse or withdraw from the interview at any time with no consequences. Only pseudonymized data were used, and transcripts were stored in secure electronic files. The study proceeded without any ethical concerns.

## Results

Expert interviews were conducted with 11 (of 11 approached) experts and 23 (of 32 approached) healthcare professionals with a median palliative care experience of 12 (range 4–25) and 8 (range 3–22) years, respectively. Reasons for declining participation were lack of time (*n* = 5), long-term sickness absence (*n* = 1), and referral to other team members (*n* = 3) (see Table 2).

**Table 2.** Sample characteristics of interviews.

Characteristics	Expert interviews *n* = 11	Episodic interviews *n* = 23
Professional background		
Physician	7	9
Nursing	2	7
Other profession	2	7
Sex		
Female	4	21
Male	7	2
Age, range in years	31–62	27–59
<30	0	1
30–40	1	5
41–50	5	8
>50	5	9
Palliative care experience in years		
3–10	4	16
11–20	3	4
>20	4	3
Care setting(s) of expertise		
Palliative care unit	5	15
Palliative care advisory team	6	11
Palliative home care team	7	7
Interview setting		
Face-to-face at workplace	3	0
Video call	8	23
Interview length, range in minutes	28–86	37–63
<45	8	12
⩾45	3	11

### Structures and processes of specialist palliative care

Participants described various consequences of the pandemic and countermeasures on specialist palliative care. Overarching aspects discussed were the increased expenditure of time caused by additional tasks like COVID testing, personnel changes and shortages due to infections within the team, and staff re-allocation. The setting itself was not per se pivotal for the extent of the described actual impact.

The superordinate goal of specialist services during the pandemic remained the provision of high-quality care. Services attempted this by troubleshooting individually arising challenges. General changes in structures and processes included using virtual communication tools, streamlining access to specialist palliative care, and obtaining special permissions for restricted processes like patient visits in hospitals.

**Figure 1.**
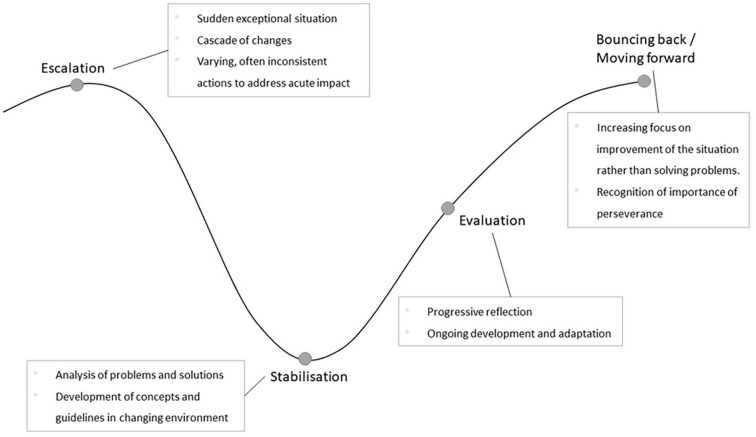
Four phases of specialist palliative care during crises.

Participant statements indicated that structural and procedural challenges during crises are deeply interconnected. The described challenges were not isolated issues but part of the complex palliative care system. It became evident that this complexity increased further during the pandemic. The findings revealed that the mechanisms and causes behind the impacts of crises is more important than an exhaustive description of individual effects. We revealed five distinct characteristics in this context: i) interconnectedness, ii) uncertainty, iii) dynamic, iv) underlying dilemmas, and v) unclear long-term goal. Furthermore, participant statements indicated four overarching, nonlinear phases in response to the challenges of the pandemic (see Figure 1).

Below, central findings are illustrated by quotes taken from the interview transcripts to ground them in the data.

#### Interconnectedness

Regarding processes and structures of specialist palliative care during the pandemic, it became evident that the connectedness and nonlinear interactions of components complicated attempts for solutions. Like a chain reaction, a problem in one area or phase of incidents might cause problems in another area. The complicating factor resulting from this was mainly the incalculable nature of emerging consequences, making it hard to anticipate.

An example were excessive contact restrictions to interrupt infection chains, causing the cancellation of team supervision in a time of increased demand for supervision. The attempt to relaunch structured supervision virtually caused new problems due to a lack of technical equipment.

(. . .) so they (the crisis committee) were convinced, at that time, that canceling meetings including supervision is absolutely necessary (. . .), we are certainly all smarter now, but back then we ended up in a really unfortunate situation because the team absolutely needed supervision. But that being said, it wasn’t easy to resolve the problem by simply running it online as we didn’t even have enough laptops, headsets and so on. (Physiotherapist, Palliative Care Unit)

#### Uncertainty

The pandemic posed a new, unfamiliar situation along with unknown problems. Hence, proven solutions for structures and processes needed to be improved and decisions on new rules developed parallel to the pandemic. Arguments had to be constantly and carefully considered and weighed up, which resulted in uncertain and unstable structural and processual regulations. Further, some extra processes caused an additional workload for the team and increased the perceived uncertainty in the context of different approaches to a solution without a clear code of practice.

A central subject described in this context were visitor restrictions on palliative care units due to infection control measures, hospital visiting restrictions, and the resulting challenges for professionals.

There is plenty of extra work for us considering visitor checks and quite some vagueness. (. . .) I have to monitor everything super detailed to make sure that we act according to the latest rules. (. . .) So in the end, we are in charge and held accountable, and it’s not always that easy because you never really know if the things you do are good enough and the way they should be. (Nurse, Palliative Care Unit)

#### Dynamic

Study participants described palliative care as a discipline well adapted to the need for high flexibility, facilitating the mastery of the pandemic. This was traced back to rapid and dynamic changes within palliative care processes regardless of the pandemic.

Finding pragmatic solutions despite the chaotic situation. That’s what I think we are very good at in palliative care anyway. Day in, day out, that’s exactly what we’re doing. And it helps to deal with COVID stuff. (Physician, Palliative Home Care Team)

However, the extremely dynamic circumstances increased the complexity of the care situation further, causing highest demands of the teams’ willingness to adopt processes and structures to secure continuity in patient care.

There is this huge bag of care needs and burden of patients and relatives. The complex trajectories we try to manage as best as possible (. . .) and then COVID hit and complicated everything even more. (. . .) As a team, you had to respond, adjust processes to engage in the original job and somehow accept the hardships. (Physician, Palliative Care Unit)

#### Underlying dilemmas

As complex systems, palliative care situations involve several individuals with different roles, which was reflected in the context of underlying dilemmas in our data. Due to powerful societal, political, and economic concerns, an additional influence on structural and processual decisions was added to the already complex situation. As the pandemic was omnipresent, processual and structural challenges affected not only patient care but also teams’ private lives, so often the borders between work and private life blurred.

Both inter- or intrapersonal dilemmas involving different interests, values, and goals emerged, for example, in challenges with the allocation of patients and discharge management during the pandemic as well as the compliance with central regulations.

On the one hand, considering taking patients back home because they are alright, on the other hand, knowing that we were already struggling with the caseload, but so was the hospital team. Having to cut down visits because of restrictions, even though we actually want to visit, because that’s how we work, that's what these patients need. But then, I wanted to end this pandemic, protect myself, and my own family at home. I had ongoing discussions in my head and often didn’t even know what I really want. (Nurse, Palliative Home Care Team)

#### Unclear long-term goal

Ultimately, it took much work to define the specific structural or processual problem resulting from the pandemic and to specify an overall long-term goal for problem management. Decisions could hence be better or worse, but not right or wrong, as the evaluation depended on the viewpoint. Traditional, analytical problem-solving following specific logical steps appeared unsuitable for the emerging challenges. Therefore, focusing on improving the situation was more productive than attempting to solve it. Accordingly, our data showed that teams benefited from agile and flexible approaches, multidisciplinary collaboration, and a high-risk tolerance within a trial-and-error mentality.

It was too complicated and too much to break it down to (. . .) plan, do, check, act, like we usually do, absolutely solution-oriented. I could not even say what the problem is, because they were all entangled like a big, damn ball of wool. So how should I tell people what they need to reconstruct, it wasn’t possible anyway. We needed new ways to cope. (Physician, expert interview on all settings)

### Mastering the pandemic: Different phases

In response to processual and structural challenges, we found that teams experienced up to four certain phases, which should be understood as a nonlinear, schematic course. A phase could occur more than once, and teams did not automatically reach every phase. Below, the four phases are presented in detail.

#### Escalation

The *escalation* phase was characterized by a sudden, unprecedented scenario, which could be the initial outbreak of COVID-19, yet equally other exceptional situations during the pandemic, such as the beginning of a new infection wave or a large number of infections within the team. Due to the unfamiliarity of the situation and because there was no prepared plan or prior experience, this phase typically led to a cascade of changes, and the resulting actions to address the acute impact were described as mainly inconsistent and unsystematic.

Suddenly all our normal processes, the usual (. . .) our usual ways of working, were interrupted (. . .) really like a true cut. And nobody had any idea or plan, so decisions were very unsteady but extremely strict. (Nurse, Palliative Care Unit)

#### Stabilization

In contrast, the *stabilization* phase was distinctly characterized by the structured analysis of problems and possible solutions to cope with newly arising conditions. Moreover, in this phase, the first concepts and guidelines were developed, and recommendations for action emerged.

We somehow muddled through it all with different ideas. When we couldn’t meet in person as a team, we discussed if videoconference, telephone, or e-mail is enough (. . .) and how we can otherwise deal with all the shifting restrictions. (Physician, Palliative Home Care Team)

#### Evaluation

The *evaluation* phase refers to increasing reflection of the situation, changing circumstances, and needs for action. Moreover, this phase was characterized by ongoing development and progressive adaptation, which showed that due to the dynamic of the pandemic, teams had to stay agile in their approaches.

(. . .) the way we did it worked well for some weeks, but then regulations changed again and again and again. You always have to reassess, reconsider and always adjust your processes, it’s like you never reach an end. (Nurse, Palliative Care Advisory Team)

It is important to note that the described phases can start over again at any point, especially in the evaluation phase. Hence, while developing a functioning solution for one problem area, a new one arises, meaning teams enter the escalation phase again.

#### Bouncing back/moving forward

By “successfully” living through the other phases, the teams could reach a fourth phase, which we identified as *bouncing back/moving forward*. This phase is characterized by an increasing focus on gradual improvement of the situation rather than attempts to solve separated issues. The team’s recognition of the importance of perseverance was a key indicator of this phase.

My understanding is that some teams wish to go back whatever may come (. . .) but that being said, some reach a point when reversing all changes isn’t the goal. Rather improving this difficult situation for all, the team, the patients, everything. And then it’s like the same but different. (. . .) They don’t know when this will be over but will hang in there. (Physician, Expert interview on all settings)

Bouncing back refers to teams aiming to restore proven, pre-crisis structures and processes, whereas moving forward promotes the long-term establishment of newly developed, functional working modalities.

## Discussion

### Main findings

This study aimed to conceptualize specialist palliative care during crises, integrating different perspectives. Our study underlines the importance of conceptually understanding the manifold effects of crises, including the interactions within the complex care situation in specialist palliative care settings. The impact of crises like the pandemic on specialist palliative care can be described through the lens of complex adaptive systems, which are integral to palliative care.^3^ Thus, the interconnectedness and interrelatedness of components, rather than isolated problems, are the essence of specialist palliative care during crises. Understanding the challenges by the characteristics i*nterconnectedness, uncertainty, dynamic, underlying dilemmas*, and *unclear long-term goal* in combination with the four portrayed phases (escalation, stabilization, evaluation, and bouncing back/moving forward) can help specialist palliative care teams, decision-makers, and policy to better understand and cope with crises.

#### What this study adds

We identified particular impacts of the pandemic and countermeasures, which is in line with previous studies showing additional workload for specialist palliative care teams,^24^ the need for adaptation of processes,^25,26^ and continuous changes influencing professionals.^4,27^

Considering the information gathered on the meso- and micro-level of care, we discovered that resolving particular problems separately is inappropriate. Instead, their interconnectedness characterizes them and combined with the changing environment of crises explains their complexity. The pandemic added an additional layer to existing complex dimensions within specialist palliative care. This partly increased conflicts of interest and impeded the rapid response capability of teams. Consequently, traditional ways of problem-solving could not address emerging issues and challenges. This fact can be transferred to other critical situations.

Structure and process characteristics of specialist palliative care during crises can be described as complex entanglement, which is equally a feature of “wicked problems,” characterized by multiple causes and interdependent factors.^28^ Notwithstanding extensive definitions for wicked problems,^28,29^ their nature can be condensed to specific key characteristics: Wicked problems are different since traditional techniques are inappropriate for solving them.^28^ Wickedness cannot be equated to the degree of difficulty, and attempts at tackling wicked problems with standard approaches may even aggravate them by causing new, negative impacts.^30^ Wicked problems are highly complex, interconnected, and hard to define. The changing and dynamic environment of wicked problems is a further quality that makes up their nature. Often, there are underlying dilemmas, like numerous stakeholders involved with different interests. Partly also strong moral or political dimensions are involved.^30,31^ The fact that the theory of wicked problems is used to describe spirals where any wrong or mistimed solution puts the problem at risk of getting worse can also be applied to specialist palliative care.

The COVID pandemic and its devastating global impact have been identified as wicked problems.^5,32^ Hence, it is not surprising that the defining characteristics and phases we identified for specialist palliative care during crises correspond reasonably well to the criteria of wicked problems (see Appendix 4). Understanding the structural and processual challenges in specialist palliative care during crises can facilitate an integrated, holistic perspective on the situation. Strengthening collaboration, trial- and error-mentality, and process-oriented approaches instead of ineffective desires to solve single problems ideally can contribute to a helpful course of action and ensure high-quality palliative care.

Reaching the state of moving forward is a progression and successful overcoming of specific problems. Hence, for specialist palliative care services, thriving through all identified phases could represent a desirable approach when dealing with future crises. By “bouncing back,” teams return to their pre-crisis structures and processes without any improvement. Moving forward, in contrast, is associated with learning and sustainable growth facilitated by overcoming crises. To support a team in reaching the state of moving forward, decision-makers and funding bodies should review, test, and consistently refine their strategy, routines, and processes as crises develop and conditions change so that teams will benefit from flexibility and agility. Understanding different phases could facilitate the efficient use and allocation of resources, enhance communication and collaboration, and identify potential for optimization. As discussed in previous literature, palliative care research requires stronger theoretical foundations to address complex care situations and improve outcomes adequately.^3^ The additional layer of complexity added by crises like a pandemic has been increasing this demand further. In this paper, we conceptualized crises and their impact on specialist palliative care services. Considering the described phases can promote a better prediction and theoretical preparedness for future crises and build a basis for actively shaping specialist palliative care services. For team management, this includes facilitating team building and providing direction, recognizing the significance of constructively managed conflicts. Furthermore, the importance of effective organization and implementing valuable feedback mechanisms should be emphasized. On the team level, approaching tasks with creativity and flexibility, and fostering an open, helpful, and collaborative relationship with colleagues are desirable.

The revealed characteristics and phases are not only applicable during major crises but universally helpful in comprehending and guiding team processes, even in the face of less severe events than a global pandemic. The extent of a crisis is, in essence, irrelevant; any challenge to routine processes might be a crisis. This can range from medication shortages, which are commonplace, to team restructuring, relocations, regulations related to assisted suicide, staff changes, and more. With an understanding of the complex dynamics within teams and the phases they undergo, interventions can be designed to prevent regression and potentially steer teams toward greater agility. Given the escalating challenges anticipated in the future, especially due to demographic shifts and the subsequent strains on the health system, it would be beneficial for teams to demonstrate maximum flexibility to adapt and evolve in response. Nevertheless, no matter how adaptable teams may be, they remain constrained if the broader framework is rigid. Therefore, the overarching health system needs to adopt flexibility, too. Eventually, all these factors directly affect the quality of care. Only with the resilience and flexibility to change, teams can handle new processes, which is crucial for providing best possible care. This is especially true when problems relate directly to care issues, such as new laws on assisted suicide, medication shortages, or changes in staff ratios, among others.

### Strengths and limitations

A notable strength of this study is the sequential approach that ensured a successive development of comprehensive results rooted in experts’ knowledge and real-world palliative care conditions. Moreover, we applied a qualitative methodology, which, by its theoretical freedom and flexibility, allowed for a rich, detailed account of data.^33^ We moved beyond the descriptive level of individual changes and conceptualized specialist palliative care during crises on an abstract level.

A limitation is that this is a national study. However, due to the focus on critical healthcare situations worldwide, the results may serve as a conceptual basis to better understand specialist palliative care during crises internationally. A further limitation is the time of data collection, which took place during the pandemic. However, by triangulating the perspective of experts with healthcare professionals’ point of view, we achieved a perception as comprehensive as possible at that time and with the resources given, providing a basis for enhancement by future studies.

## Conclusion

Understanding the impact of crises on specialist palliative care as a highly complex problem, causing challenges but also opportunities, is important to address possible future crises. A fundamental understanding of team complexity must be considered in the context of leadership as well as team development at all times. This insight is vital for effective management and fostering team growth. The described concept can promote a holistic, open approach and the inclusion of different stakeholders in decision-making. This demands high degrees of interdisciplinary collaboration. It is necessary to explore further the theory and potential adaptations to other healthcare systems. This will be particularly important as crises will remain a persistent thread for specialist palliative care, and plans need to comprise lessons learned. In summary, addressing complex challenges caused by crises requires system thinking and a learning mindset, facilitating teams to overcome critical situations and move forward rather than bounce back to normal.

## Figures and Tables

**Figure A1:**
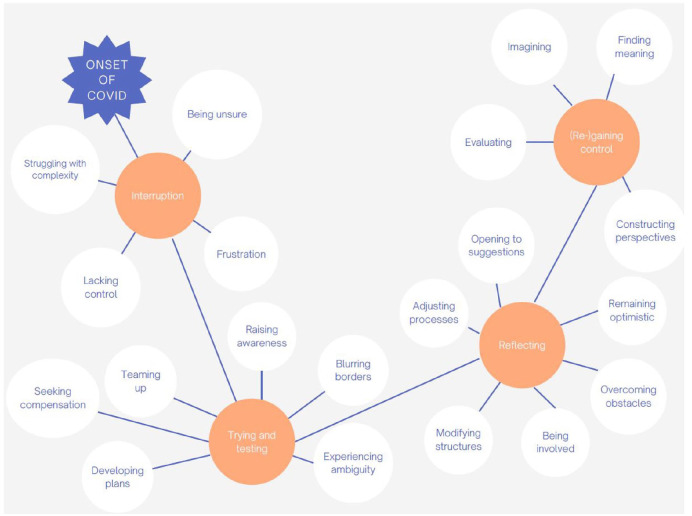
Code tree.

## Appendix 1

**Table A1.** Questions on COVID within expert interviews^a^.

Guiding question	Clarifying questions	Prompts
When you think back to how structures and processes in palliative care were shaped before the outbreak of the pandemic and how it is now—since the spread of COVID-19—what has changed on the level of structure and process characteristics of specialist palliative care as a result of the pandemic situation? Which of the characteristics you addressed in the general context are particularly affected by the impact of the pandemic from your point of view?	To what extent does the impact of the pandemic to date differ between different types of specialist palliative care teams? What are differences regarding the impact of the pandemic so far between different types of specialist palliative care teams in terms of processes? What potential impact within specialist palliative care do you think is likely in the context of the long-term development of the pandemic?	Could you give further details on this? Could you please elaborate on this? Could you give an example? Is there anything else you want to mention? Why do you think that is?

a For full topic guide see Wikert et al.^12^

## Appendix 2

**Table A2.** Topic guide for episodic interviews.

Guiding questions	Clarifying questions	Prompts
Introducing question: First, what are your associations with the COVID pandemic?		Could you give further details on this? Could you please elaborate on this? Could you give an example? Is there anything else you want to mention? Why do you think that is?
Part 1: Outbreak of the pandemic Please remember the moment when you first learned about the COVID-19 pandemic: How did you get to know about the outbreak? Could you please tell me about the situation?	To what extent did the professional context play a role: ● Point of time ● Manner (e.g., worries, fears, challenges, ideas) ● Professional interaction/exchange (with colleagues; in meetings, planning)	
Part 2: Impact on specialist palliative care Next, I would like to ask you to tell me what changes the pandemic has brought about in your everyday work in your work setting(s). Can you tell me about a situation that makes this clear?	To what extent are there differences in terms of structures and processes, for example: - General condition - Workflows, routine - Particular procedures (focus, new, discontinued, etc.)	
Part 3: Semantic knowledge Now, after we have spent some time talking about connections between the pandemic and the daily professional care you provide, I would like to switch to a different aspect: What is particularly important to you at work? / What do you place special emphasis on? Which aspects of this are particularly affected by the pandemic?	To be considered: - Values - (Perceived) possibility of influencing the situation To what extent are there specifics in the context of palliative care compared to other areas of healthcare (and during the pandemic)? Which longer-term consequences are expected?	

## Appendix 4

### Characteristics of wicked problems

1. There is no definitive formulation of a wicked problem.
2. Wicked problems have no stopping rule.
3. Solutions to wicked problems are not true-or-false, but good-or-bad.
4. There is no immediate and no ultimate test of a solution to a wicked problem.
5. Every solution to a wicked problem is a “one-shot operation”; because there is no opportunity to learn by trial-and-error, every attempt counts significantly.
6. Wicked problems do not have an enumerable (or exhaustively desirable) set of potential solutions, nor is there a well-described set of permissible operations that may be incorporated into the plan.
7. Every wicked problem is essentially unique.
8. Every wicked problem can be considered to be a symptom of another problem.
9. The existence of a discrepancy representing a wicked problem can be explained in numerous ways. The choice of explanation determines the nature of the problem’s resolution.
10. The planner has no right to be wrong.
